# Correcting calculation and data errors reveals that the original conclusions were incorrect in “The best drug supplement for obesity treatment: a systematic review and network meta-analysis”

**DOI:** 10.1186/s13098-023-01134-6

**Published:** 2023-07-22

**Authors:** Xiaoxin Yu, Patrice L. Capers, Roger S. Zoh, David B. Allison

**Affiliations:** 1 Department of Epidemiology and Biostatistics, School of Public Health, Indiana University – Bloomington, 1025 E. 7th St, Bloomington, IN 47405 USA; 2 Department of Biology, Swain Family School of Science and Mathematics, The Citadel – Charleston, Charleston, SC USA

**Keywords:** Network meta-analysis, Drug supplement, Obesity treatment, Rigor, Reproducibility

## Abstract

The goal of this study was to reproduce and evaluate the reliability of the network meta-analysis performed in the article “The best drug supplement for obesity treatment: A systematic review and network meta-analysis” by Salari et al. In recent years, it has become more common to employ network meta-analysis to assess the relative efficacy of treatments often used in clinical practice. To duplicate Salari et al.‘s research, we pulled data directly from the original trials and used Cohen’s D to determine the effect size for each treatment. We reanalyzed the data since we discovered significant differences between the data we retrieved and the data given by Salari et al. We present new effect size estimates for each therapy and conclude that the prior findings were somewhat erroneous. Our findings highlight the importance of ensuring the accuracy of network meta-analyses to determine the quality and strength of existing evidence.

## Introduction

In the publication by Salari et al. [1], “The best drug supplement for obesity treatment: A systematic review and network meta-analysis,” the authors used a network meta-analysis to analyze the effectiveness of several anti-obesity medications. In the history of obesity pharmacotherapy, several promising treatment candidates have arisen only to be discontinued owing to unacceptably high safety concerns [1]. The potential value of obesity pharmacotherapy encourages the publication of meta-analysis papers that combine and rigorously compare studies to determine the quality and strength of the existing evidence.

**Table 1:** Re-extracted information on mean age and treatment alongside data reported in the Salari et al. paper

				Age (y; Salari Paper)		Age (y; New Results^a^)	
Study	Year	Groups (Salari Paper)	Groups (New Results^a^)	Mean	SD	Mean	SD
Apovian [9]	2013	Placebo	Placebo	44.4	11.4	44.4	11.4
		Naltrexone + bupropion	32 mg/day naltrexone SR + 360 mg/day bupropion SR (NB32)	44.3	11.2	44.3	11.2
Aronne [10]	2010	Placebo	Placebo	42	11	42	11
		Pramlintide	Pramlintide (120 µg sc)	42	11	42	11
			Pramlintide (120 µg sc t.i.d.) + sibutramine (10 mg oral q.a.m.)			43	11
			Pramlintide (120 µg sc t.i.d.) + phentermine (37.5 mg oral q.a.m.)			39	10
Davies [7]	2015	Placebo	Placebo	54.7	9.8	54.7	9.8
		Liraglutide 0.3 mg	Liraglutide 3.0 mg			55	10.8
		Liraglutide 1.8 mg	Liraglutide 1.8 mg			54.9	10.7
Fidler [11]	2011	Placebo	Placebo	43.7	11.8	43.7	11.8
		Lorcaserin 10 mg BID	Lorcaserin 10 mg BID	43.8	11.8	43.8	11.8
		Lorcaserin 10 mg QD	Lorcaserin 10 mg QD	43.8	11.7	43.8	11.7
Gadde [12]	2011	Placebo	Placebo	51.2	10.25	51.2	10.25
		Phentermine 7.5 mg + topiramate 46.0 mg	Phentermine 7.5 mg + topiramate 46.0 mg	51.1	10.43	51.1	10.43
		Phentermine 15.0 mg + topiramate 92.0 mg	Phentermine 15.0 mg + topiramate 92.0 mg	51	10.65	51	10.65
Greenway [8]	2010	Placebo	Placebo	43.7	11.1	43.7	11.1
		Naltrexone + bupropion 16.0 mg	Naltrexone16.0 mg + bupropion	44.4	11.3	44.4	11.3
		Naltrexone + bupropion 32.0 mg	Naltrexone32.0 mg + bupropion	44.4	11.1	44.4	11.1
Le Roux [13]	2017	Placebo	Placebo	47.3	11.8	47.3	11.8
		Liraglutide	Liraglutide 3.0 mg	47.5	11.7	47.5	11.7
Lu [14]	2018	Placebo	Placebo	37	10	37	10
		Lorcaserin	Lorcaserin 10.0 mg	34.7	9	34.7	9
O’Neil [15]	2012	Placebo	Placebo	53.2	8.3	52	9.3
		Orlistat 120.0 mg BID	Lorcaserin 10 mg BID	53.9	8.1	53.2	8.3
		Orlistat 120.0 mg QD	Lorcaserin 10 mg QD	53.5	7.4	53.1	8
Pi-Sunyer [16]	2015	Placebo	Placebo	45.2	12.1	45	12
		Liraglutide	Liraglutide 3.0 mg QD	45	12	45.2	12.1
Smith [17]	2010	Placebo	Placebo	44.4	0.3	44.4	0.3
		Lorcaserin	Lorcaserin 10.0 mg	43.8	0.3	43.8	0.3

Abbreviations: BID, twice a day; q.a.m., every morning; QD, every day; sc, subcutaneous; t.i.d., three times a day

The difference between our extraction and original study used underlining

a: “New” results are from our data extraction from the original studies. Underlining denotes discrepant values

A network meta-analysis uses both direct and indirect data from a network of trials to compare three or more treatments at once in a single study. The use of network meta-analysis to evaluate the relative efficacy of therapies often used in clinical practice has gained popularity recently [3]. There are several advantages to using network data for meta-analytical purposes, including deriving more exact estimations of the relative impact of each intervention in the network. Additionally, using network meta-analysis allows investigators to rank the interventions included in the analysis [4]. Credible inference in a network meta-analysis is premised on the assumption that the different studies included in the analysis are similar in terms of all major features that might affect the relative effects [5] and that the analysis was properly conducted. It is therefore essential to ensure that meta-analyses and network meta-analyses are conducted correctly.

Upon inspection of Salari’s et al. paper, we uncovered several data reporting and extraction errors that render the results invalid. We brought these errors to the attention of the authors, leading them to publish a correction [6], but the correction did not address all the errors that we discussed. The correction addressed the following: in Table 1, the treatment reported for the Davies et al. study [7] was incorrect (liraglutide 0.3 mg should be liraglutide 3.0 mg) and the wrong supplement was reported for the Greenway et al. study [8] (naltrexone + bupropion 16.0 mg and naltrexone + bupropion 32.0 mg; should be naltrexone 16.0 mg + bupropion and naltrexone 32.0 mg + bupropion).

Because the correction issued by the authors did not address all the errors we previously identified, we redid the network meta-analysis. Note that the analysis by Salari et al. involved 11 parallel studies which we refer to as the “original studies”: Apovian et al. [9], Aronne et al. [10], Davies et al. [7], Fidler et al. [11], Gadde et al. [12], Greenway et al. [8], Le Roux et al. [13], Lu et al. [14], O’Neil et al. [15], Pi-Sunyer et al. [16], and Smith et al. [17]. Here we report the discrepancies between what Salari et al. reported and what we obtained by extracting data directly from the original studies.

## Methods

### Data extraction and evaluation

We first attempted to collect the original datasets and code from the corresponding author. We reached out to the corresponding author of the original research manuscript on October 27, 2021, asking them to share their data and the R code used to generate their results. Dr. Mohammadi provided us with two materials: the appendix of the preliminary results (a Microsoft Excel file) and an R script file. Salari et al. carried out their systematic review and network meta-analysis, which we refer to as the “original research”, by conducting a systematic database search, categorizing documents for evaluation, applying inclusion and exclusion criteria, extracting data, and conducting the network meta-analysis. The data reported in the original research paper were from participants who completed post-treatment assessments.

We sought to recapitulate this analysis by extracting the same data from completers in the 11 original studies. We extracted data (sample size, mean, standard deviation, treatment name, etc.) directly from the original studies listed in Tables 1 and 2 of the Salari et al. paper. The sample size, mean, and standard deviation refer to each arm of every study. The data extracted were verified by two or more researchers.

**Table 2:** Re-extracted information on sex and number of participants alongside data reported in the Salari et al. paper

				Salari Paper Results (N)			New Results^a^ (N)		
Study	Year	Groups (Salari Paper)	Groups (New Results^a^)	Total Patients	Men (N)	Women (N)	Total Patients	Men (N)	Women (N)
Apovian [9]	2013	Placebo	Placebo	1496	76	419	1496	76	419
		Naltrexone + bupropion	32 mg/day naltrexone SR + 360 mg/day bupropion SR (NB32)		155	846		155	846
Aronne [10]	2010	Placebo	Placebo	244	13	87	244	8	55
		Pramlintide	Pramlintide (120 µg sc)		12	88		7	54
			Pramlintide (120 µg sc t.i.d.) + sibutramine (10 mg oral q.a.m.)					6	53
			Pramlintide (120 µg sc t.i.d.) + phentermine (37.5 mg oral q.a.m.)					8	53
Davies [17]	2015	Placebo	Placebo	864	97	115	846	97	115
		Liraglutide 0.3 mg	Liraglutide 3.0 mg		220	203		220	203
		Liraglutide 1.8 mg	Liraglutide 1.8 mg		108	103		108	103
Fidler [11]	2011	Placebo	Placebo	4004	353	1248	4004	353	1248
		Lorcaserin 10 mg BID	Lorcaserin 10 mg BID		313	1289		313	1289
		Lorcaserin 10 mg QD	Lorcaserin 10 mg QD		145	656		145	656
Gadde [12]	2011	Placebo	Placebo	2487	299	695	2487	299	695
		Phentermine 7.5 mg + topiramate 46.0 mg	Phentermine 7.5 mg + topiramate 46.0 mg		149	349		149	349
		Phentermine 15.0 mg + topiramate 92.0 mg	Phentermine 15.0 mg + topiramate 92.0 mg		302	693		302	693
Greenway [8]	2010	Placebo	Placebo	1742	85	496	1742	85	496
		Naltrexone + bupropion 16.0 mg	Naltrexone16.0 mg + bupropion		88	490		88	490
		Naltrexone + bupropion 32.0 mg	Naltrexone32.0 mg + bupropion		87	496		87	496
Le Roux [13]	2017	Placebo	Placebo	2254	176	573	2254	176	573
		Liraglutide	Liraglutide 3.0 mg		364	1141		364	1141
Lu [14]	2018	Placebo	Placebo	171	28	57	170	28	57
		Lorcaserin	Lorcaserin 10.0 mg		39	46		39	46
O’Neil [15]	2012	Placebo	Placebo	508	73	84	603	115	137
		Orlistat 120.0 mg BID	Lorcaserin 10 mg BID		86	83		119	137
		Orlistat 120.0 mg QD	Lorcaserin 10 mg QD		34	41		42	53
Pi-Sunyer [16]	2015	Placebo	Placebo	3731	273	971	3731	273	971
		Liraglutide	Liraglutide 3.0 mg QD		530	1957		530	1957
Smith [17]	2010	Placebo	Placebo	3182	253	1331	3182	253	1331
		Lorcaserin	Lorcaserin 10.0 mg		272	1321		272	1321

Abbreviations: BID, twice a day; q.a.m., every morning; QD, every day; sc, subcutaneous; t.i.d., three times a day

The difference between our extraction and original study used underlining

a: “New” results are from our data extraction from the original studies. Underlining denotes discrepant values

### Analysis

We used the extracted data to calculate the effect size for each treatment using Cohen’s D metric to estimate the weight difference between groups due to changes from baseline. Upon review of the original studies, however, we found that the data in some studies were reported differently from what Salari et al. stated in their original publication^1^ and in the correction [6]. Some of the original studies did not report the actual final weight value but instead reported the mean change in weight in kilograms (kg). We thus calculated the final weight values or mean change in weight for both the original studies and number reported by Salari et al. Similarly, we computed the number of participations based on the sex distribution (percentage) reported in the original studies. We then recapitulated the network meta-analysis in R studio using the data we extracted to explore the most effective drug treatment for obesity. We compared our results with the data provided by the corresponding author and the data reported by Salari et al. in their original research.

## Results

**Fig. 1:**
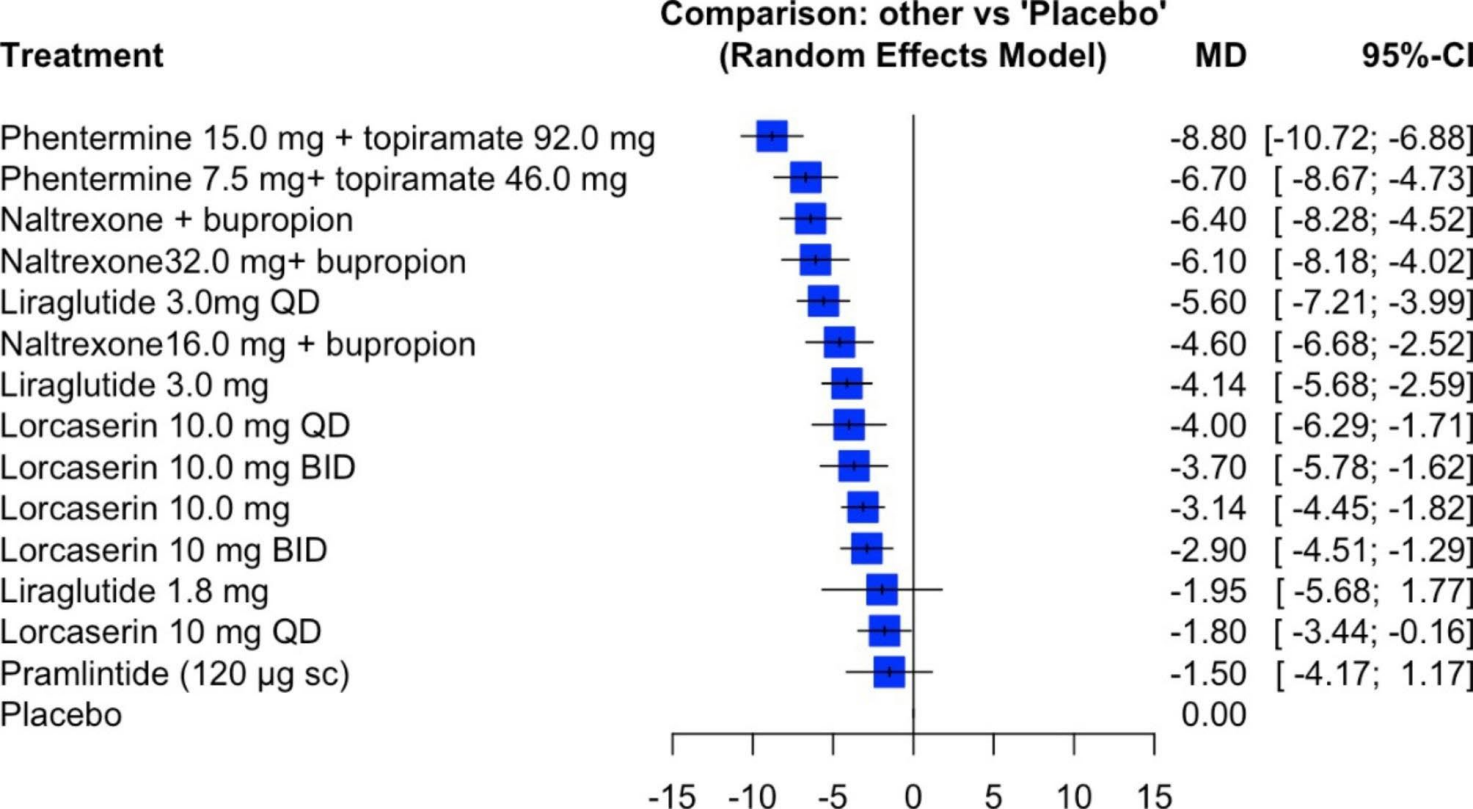
Meta-analysis study of various drug supplements used in the treatment of obesity using re-extracted values based on a random-effects model

In Table 1 (age and treatment), Table 2 (sex and number of participants), and Table 3 (weight and weight change), we compare the data we extracted from the original studies with those reported by Salari et al. Our new estimates of the effect sizes are included in Fig. 1.

**Table 3:** Re-extracted information on initial mean weight, final weight, and mean weight change

			Initial Weight (kg)		Final Weight (kg)	Mean Weight Change (kg)			
**Study**	**Year**	**Groups**	**Mean**	**SD**	**Mean**	**Mean**	**SE**	**SD**	**N** ^a^
Apovian [9]	2013	Placebo	99.2	15.9	97.7	-1.5	0.5	8.17	267
		32 mg/ day naltrexone SR + 360 mg/day bupropion SR (NB32)	100.3	16.6	92.4	-7.9	0.3	6.25	434
Aronne [10]	2010	Placebo	107	22	104.9	-2.1	0.9	6.85	58
		Pramlintide (120 µg sc)	102	19	98.4	-3.6	0.7	5.33	58
		*Pramlintide (120 µg sc t.i.d.) + sibutramine (10 mg oral q.a.m.)	101 (NA)	16 (NA)	89.7 (NA)	-11.3 (NA)	1.2 (NA)	9.06 (NA)	57
		*Pramlintide (120 µg sc t.i.d.) + phentermine (37.5 mg oral q.a.m.)	102 (NA)	18 (NA)	90.7 (NA)	-11.3 (NA)	0.9 (NA)	6.91 (NA)	59
Davies [7]	2015	*Placebo	106.5	21.3	101.3 (104.3)	-5.2 (-2.2)	1.44 (NA)	20.86 (NA)	211
		*Liraglutide 3.0 mg	105.7	21.9	98.2 (99.3)	-7.5 (-6.4)	1.04 (NA)	21.13 (NA)	412
		*Liraglutide 1.8 mg	105.8	21	99.8 (100.8)	-6 (-5)	1.47 (NA)	21.05 (NA)	204
Fidler [11]	2011	Placebo	100.8	16.2	97.9	-2.9	0.163 (NA)	6.4	1541
		Lorcaserin 10 mg BID	100.3	15.7	94.5	-5.8	0.162 (NA)	6.4	1561
		Lorcaserin 10 mg QD	100.1	16.7	95.4	-4.7	0.23 (NA)	6.4	771
Gadde [12]	2011	Placebo	103.3	18.1	101.9	-1.4	0.36 (NA)	11.2 (NA)	979
		Phentermine 7.5 mg + topiramate 46.0 mg	102.6	18.2	94.5	-8.1	0.51 (NA)	11.3 (NA)	488
		*Phentermine 15.0 mg + topiramate 92.0 mg	103.3 (103)	17.6	93.1 (92.8)	-10.2	0.46 (NA)	14.4 (NA)	981
Greenway [8]	2010	Placebo	99.5	14.3	97.6	-1.9	0.5	8.5 (NA)	290
		Naltrexone16.0 mg + bupropion	99.5	14.8	93	-6.5	0.5	8.4 (NA)	284
		Naltrexone32.0 mg + bupropion	99.7	15.9	91.7	-8	0.5	8.6 (NA)	296
Le Roux [13]	2017	Placebo	107.9	21.8	105.9	-2	0.27 (NA)	7.3	738
		Liraglutide 3.0 mg	107.5	21.6	101	-6.5	0.21 (NA)	8.1	1472
Lu [14]	2018	Placebo	91.5	14.5	87.9	-3.6	0.65 (NA)	5.87 (NA)	82
		Lorcaserin 10.0 mg	92.6	13.3	86.8	-5.8	0.56 (NA)	5.14 (NA)	84
O’Neil [15]	2012	*Placebo	101.6	18.1	99.7 (101.7)	-1.9 (NA)	0.5 (NA)	6.26 (NA)	157
		*Lorcaserin 10 mg BID	104.7	17.9	99.1 (104.7)	-5.6 (NA)	0.5 (NA)	6.50 (NA)	169
		*Lorcaserin 10 mg QD	105.4 (105.9)	19	99.5 (105.4)	-5.9 (NA)	0.7 (NA)	6.06 (NA)	75
Pi-Sunyer [16]	2015	Placebo	106.2	21.7	103.4	-2.8	0.19 (NA)	6.5	1225
		Liraglutide 3.0 mg QD	106.2	21.2	97.8	-8.4	0.15 (NA)	7.3	2437
Smith [17]	2010	Placebo	99.7	15.93 (0.4)	97.5	-2.2	0.1	3.87 (NA)	1499
		Lorcaserin 10.0 mg	100.4	15.97 (0.4)	94.6	-5.8	0.2	7.84 (NA)	1538

Original results reported in Salari et al.’s paper^1^ are shown in parentheses. Discrepant results are shown in red. Abbreviations: BID, twice a day; q.a.m., every morning; QD, every day; sc, subcutaneous; t.i.d., three times a day

a: The number of participants is from completers

*: The treatment groups from which the data were re-extracted and/or calculated differed from those reported by Salari et al

We found several discrepancies between the data we extracted and the data in the spreadsheet we received from the corresponding author. For example, their dataset did not include all the studies listed in the article as having been analyzed. Specifically, Aronne et al.’s study, Le Roux et al.’s study, and Lu et al.’s study were completely missing from the dataset but were reported in the published paper.

When we carefully reviewed the information reported in Salari et al.’s Tables 1 and 2 and compared it with the information reported in the original studies, we found several data extraction errors or discrepancies. For example, in Table 1 of the original research paper, the mean age and standard deviation are completely missing for the Davies et al. study in the interventions column [7]. However, these data were reported in the original study by Davies et al.: the mean (SD) ages were 55 (10.8) years in the liraglutide 3.0 mg group and 54.9 (10.7) years in the liraglutide 1.8 mg group. We also found data extraction errors for initial average weight and average weight change in Table 2 of the Salari et al. paper. Salari et al. did not disclose whether the variance reported (e.g.: ± 6.4 in Fidler et al.’s study) in Table 2 was a standard deviation or a standard error. Standard deviation of weight change is used to calculate the treatment effect standard error. If the authors reported the treatment effect estimates and their uncertainty using standard error for some and standard deviation for others, these effects would not be directly comparable. They should not be directly used as input for the network. We found that some of the original studies used standard deviation, and other used standard error.

**Fig. 2:**
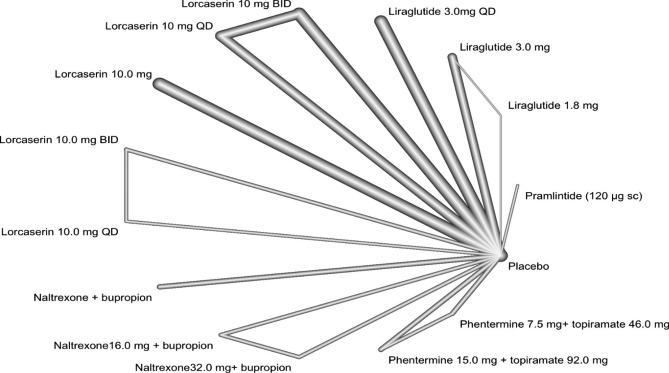
The final network diagram created from the re-extracted values

We believe the aforementioned discrepancies and errors in addition to the others reported in Tables 1, 2 and 3 influenced the results of the network meta-analysis performed by Salari et al. After we calculated the differences in weight loss between each drug vs. placebo and conducted a random-effects model, we arrived at different values for the effect size (and 95% CI) compared with those reported in Fig. 6 of Salari et al. Salari et al. claimed to show the standardized mean difference (SMD). However, they reported mean differences between the groups that differed from our recapitulated results (Figs. 1 and 2) using the re-extracted values. For example, the effect size of phentermine 15.0 mg + topiramate 92.0 mg should be -8.8 [-10.72, -6.88], not − 9.10 [-10.37, -7.83], and the effect size of pramlintide should be -1.5 [-4.17, 1.17], not − 6.50 [-13.46, 0.46].

## Discussion and conclusion

According to the Committee on Publication Ethics’ Retraction Guidelines, retraction should be considered if there is “clear evidence that the findings are unreliable… as a result of a major error (e.g., miscalculation or experimental error)” [18]. When the errors in the paper by Salari et al. are corrected, we find a substantially different rank order of drugs in terms of the most effective weight-loss medications. We respectfully believe that Salari et al.’s network meta-analysis should be retracted because the conclusion drawn is inaccurate owing to miscalculation and inaccuracy of the data reported.

Additionally, as one reviewer noted, “It is also problematic that only completers were analyzed. A sensitivity analysis using the pattern-mixture model to model the missing participants would have elucidated whether missingness may threaten the validity of the results.” [19]. We agree. Our purpose herein was to evaluate whether the results could be reproduced (as defined by the National Academy of Sciences [20], They could not be. Future research should determine the answers obtained when the analyses are conducted optimally. Obtaining such answers will require a new full-scale endeavor including sensitivity analyses to respond to concerns around treatment of missing data.

## Data Availability

Analysis data may be obtained from the corresponding author at allison@iu.edu.
